# Exposure of Neonatal Mice to Tobacco Smoke Disturbs Synaptic Proteins and Spatial Learning and Memory from Late Infancy to Early Adulthood

**DOI:** 10.1371/journal.pone.0136399

**Published:** 2015-08-25

**Authors:** Larissa Helena Torres, Raphael C. T. Garcia, Anne M. M. Blois, Lívia M. M. Dati, Ana Carolina Durão, Adilson Silva Alves, Maurílio Pacheco-Neto, Thais Mauad, Luiz R. G. Britto, Gilberto Fernando Xavier, Rosana Camarini, Tania Marcourakis

**Affiliations:** 1 Department of Clinical and Toxicological Analysis, School of Pharmaceutical Sciences, University of São Paulo, São Paulo/SP, CEP: 05508–000, Brazil; 2 Department of Physiology and Biophysics, Institute of Biomedical Sciences, University of São Paulo, São Paulo/SP, CEP: 05508–000, Brazil; 3 Department of Clinical Pathology, School of Medicine, University of São Paulo, São Paulo/SP, CEP: 05403–010, Brazil; 4 Department of Pathology, School of Medicine, University of São Paulo, São Paulo/SP, CEP: 01246–903, Brazil; 5 Department of Physiology, Institute of Biosciences, University of São Paulo, São Paulo/SP, CEP: 05508–900, Brazil; 6 Department of Pharmacology, Institute of Biomedical Sciences, University of São Paulo, São Paulo/SP, CEP: 05508–900, Brazil; Universidade do Estado do Rio de Janeiro, BRAZIL

## Abstract

Exposure to environmental tobacco smoke (ETS) in the early postnatal period has been associated with several diseases; however, little is known about the brain effects of ETS exposure during this critical developmental period or the long-term consequences of this exposure. This study investigated the effects of the early postnatal ETS exposure on both reference and working memory, synaptic proteins and BDNF from late infancy to early adulthood (P3-P73). BALB/c mice were exposed to ETS generated from 3R4F reference research cigarettes (0.73 mg of nicotine/cigarette) from P3 to P14. Spatial reference and working memory were evaluated in the Morris water maze during infancy (P20-P29), adolescence (P37-P42) and adulthood (P67-P72). Synapsin, synaptophysin, PSD95 and brain-derived neurotrophic factor (BDNF) were assessed at P15, P35 and P65 by immunohistochemistry and immunoblotting. Mice that were exposed to ETS during the early postnatal period showed poorer performance in the spatial reference memory task. Specifically, the ETS-exposed mice exhibited a significantly reduced time and distance traveled in the target quadrant and in the platform location area than the controls at all ages evaluated. In the spatial working memory task, ETS disrupted the maintenance but not the acquisition of the critical spatial information in both infancy and adolescence. ETS also induced changes in synaptic components, including decreases in synapsin, synaptophysin, PSD95 and BDNF levels in the hippocampus. Exposure to ETS in the early postnatal period disrupts both spatial reference and working memory; these results may be related to changes in synaptogenesis in the hippocampus. Importantly, most of these effects were not reversed even after a long exposure-free period.

## Introduction

Tobacco is the most frequently used licit drug among pregnant women around the world. In North America, the use of tobacco during pregnancy is approximately 18% [1], and in countries such as the Netherlands, Turkey, Morocco, Surinam, Cape Verde and Antilles, its use among pregnant women reaches 30% [2].

Passive smokers are exposed to environmental tobacco smoke (ETS), one of the most common indoor pollutants, which affects approximately 40% of children, 35% of women, and 33% of men [3]. During childhood, ETS is associated with cognitive and neurobehavioral impairments, including depression, impulsivity, attention-deficit hyperactivity disorder, conduct disorders and drug abuse [4]. In rodents, exposure to ETS during adulthood has been shown to induce apoptotic cell death [5] and reactive astrogliosis [6]. However, few studies have evaluated the effects of ETS on brain development. In addition, most of these studies do not focus on processes that are considered to be critical at this period [7–10]. In the hippocampus, the maturation of excitatory synaptic transmission, which takes place around the second postnatal week, is required for the expression of cognitive processes, such as learning and memory, in adults [11].

The early weeks of the postnatal period involve rapid brain growth, with peak synaptogenesis, gliogenesis, and maturation of neurotransmission, all of which are influenced by environmental stimuli such as nicotine and ETS [12–15]. Exposure to nicotine can disturb brain development due to the critical role of acetylcholine during this period. The transient upregulation of nicotinic acetylcholine receptors (nAChR) in the critical phases of several brain structures highlights the importance of the cholinergic system during development [14]. Postnatal exposure to sidestream smoke induced a decrease in the total number of cells and an increase in the cell size in the hindbrain of rats [16]. Non-human primates exposed to ETS during the perinatal period show upregulation of nAChRs and the serotonin 1A receptor (5HT1A), suggesting that ETS can disturb the development of the cholinergic and serotoninergic systems [17,18]. However, it is not known whether such changes lead to long-term morphological and biochemical changes in pre-synaptic elements or deficits in learning and memory. Thus, the aim of the present study was to investigate the effects of ETS in the early postnatal period in synaptogenesis, learning and memory and its possible consequences in adolescence and early adulthood.

## Materials and Methods

### Animals

One hundred twenty-four BALB/c mice pups from 20 litters were used in the biochemical and behavioral tests [Immunoblotting and BDNF: 36 animals, 6 litters; immunohistochemistry: 30 animals, 6 litters; exposure biomarkers: 10 animals, 10 litters (same litters as the immunoblotting and immunohistochemistry); Morris water maze: 48 animals, 8 litters]. Two animals died before the procedures were complete. The animals were obtained from the animal facility of the University of São Paulo, School of Medicine. All animals were housed at 20–22°C with a 12 h/12 h light/dark cycle (lights on at 07:00 a.m.) and received water and commercial food pellets for small rodents from Nuvital (Nuvilab CR-1; Colombo, Brazil) *ad libitum*.

### Ethics statement

All procedures were approved by the Ethics Committee of the School of Medicine (1038/09), the Institute of Biomedical Science (83-111-02/11) and the School of Pharmaceutical Sciences (260/10) of the University of São Paulo.

### Experimental design

**Fig 1:**
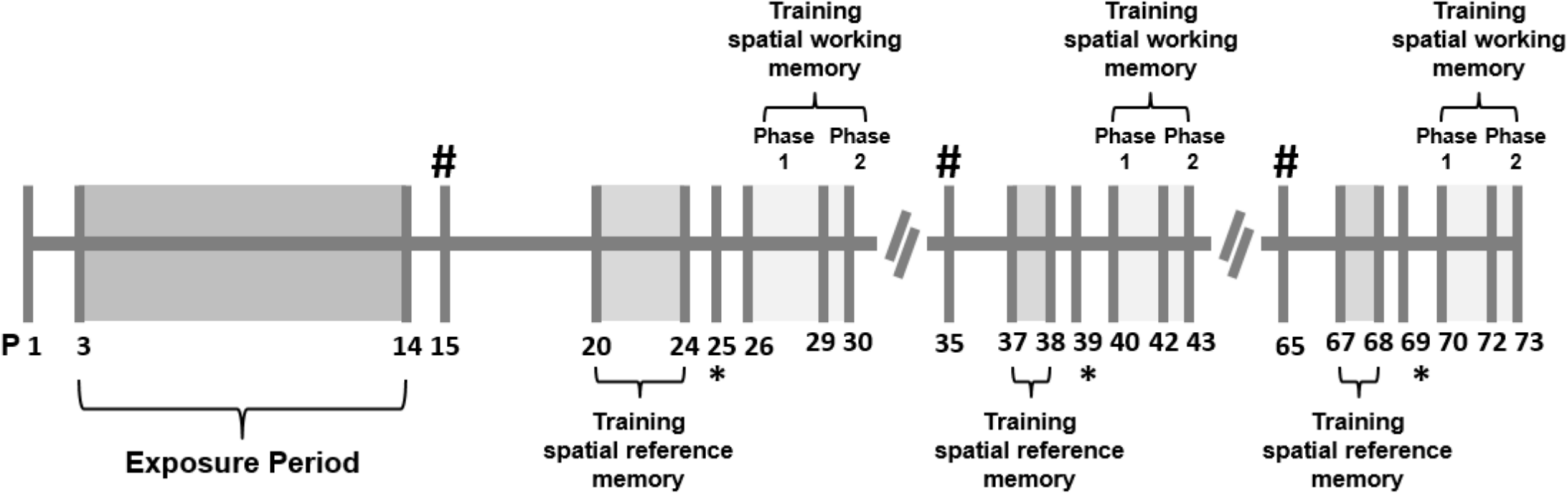
Summary of the experimental design. The animals were exposed to a mixture of mainstream and sidestream tobacco smoke from the reference cigarettes 3R4F from P3 to P14. For the immunoblotting and immunohistochemistry analyses, the animals were euthanized (#) at P15 (infancy), P35 (adolescence) or P65 (adulthood). A spatial reference memory training task in the Morris water maze was performed at P20-P24 (infancy), P37-P38 (adolescence) and P67-P68 (adulthood), with probe tests (*) at P25, P39 and P69. The spatial working memory task involved two phases. In Phase 1 (at P26-P29, P40-P42, and P70-P72), the intertrial interval was 10 min; in Phase 2 (P30, P43 and P73), the intertrial interval was reduced to 0 min. See the main text for details.

A summary of the experimental schedule is shown in Fig 1.

Within the first day after delivery, animals born on the same day were randomly distributed to ensure that each litter had a representation of the original litter and guarantee 6–7 pups per litter. The small weight variation within each group indicates that this procedure did not influence the breeding (data not shown). For each experiment, 1 to 2 animals from different litters were used to compose a group. BALB/c pups were exposed to ETS as previously described [9,10]. Briefly, pups and dams were subjected to two 1-h exposures per day (1 h at 8 a.m. and 1 h at 5 p.m.), to a mixture of mainstream and sidestream tobacco smoke from reference 3R4F cigarettes (0.73 mg of nicotine/cigarette; College of Agriculture, University of Kentucky) from the postnatal day 3 (P3) to 14 (P14). The exposures took place within a chamber measuring 564x385x371 mm. The level of carbon monoxide (CO) in the chamber was measured every 10 minutes using a gas detector (ToxiPro, Biosystems, USA). Control animals were exposed to the same experimental conditions but inhaled only air delivered into the chamber from an air cylinder at 2 mL/min (pressure: 4 bar). Immediately after the last exposure, the animals (*n* = 5) were anesthetized and the blood was collected by transcardiac puncture to quantify the biological exposure markers. Carboxyhemoglobin (COHb) was analyzed using the spectrophotometric method, and plasma nicotine and cotinine were measured by gas chromatography with a nitrogen/phosphorus detector [9,10].

Different sets of both male and female pups were randomly assigned to three groups: P15 (infancy), P35 (adolescence) and P65 (adulthood) for immunoblotting (*n* = 6; 3 females and 3 males), immunohistochemistry (*n* = 5; 2 females and 3 males) and the BDNF (*n* = 6; 3 females and 3 males) assays. Twenty-four hours after the last smoke exposure (P15) or at P35 or P65, the animals were anesthetized and perfused for immunohistochemistry or euthanized by cervical dislocation for immunoblotting and BDNF assays. The brains were removed, and the hippocampi were dissected and stored at -80°C until the homogenates were prepared. For the behavioral tests, forty-eight animals (24 females and 24 males) were used from P20 to P73 to evaluate whether the effects of ETS exposure in the early postnatal period are persistent and if they disrupt learning and memory processes from infancy to early adulthood.

### Morris water maze

The Morris water maze, a circular pool (90 cm in diameter, 50 cm in height) filled to a depth of 40 cm with water (23°C ± 1) and rendered opaque by the addition of milk, was located in a room with several salient cues hanging on the walls. A movable transparent circular acrylic platform of 9 cm in diameter was placed in the pool 1 cm below the water surface. In each trial, the starting position was changed randomly, and the mice were individually allowed to swim freely for a maximum of 60 s. Once the mouse found the platform, it was permitted to remain on it for 10 s. The animals that did not find the platform were gently guided to it and placed on it for 10 s. The water maze was divided into four equal-area quadrants, and the platform location was either maintained in a fixed position or varied depending on the behavioral testing procedure (see below). Each trial was recorded with a digital camera, and the activity analysis was performed with the behavioral tracking system EthoVision (EthoVision 3.1 software; Noldus, Wageningen, the Netherlands). Given the sex differences observed in preclinical studies that focus on nicotine exposure during brain development, male and female mice were used in behavioral tests [19–21]. The Morris water maze was used to evaluate spatial reference and working memory, as described below.

#### Spatial reference memory

In the spatial reference memory task, the platform location remained constant throughout the training in the center of the northwest quadrant. During infancy, each animal performed four trials per session, one session per day, for five days (P20 to P24) (*n* = 12). The intertrial interval (ITI) was 1 minute. During adolescence and adulthood stages (P37 to P38 and P67 to P68, respectively), the training sessions were reduced to two days because the animals had already performed the task during infancy. The performance in the reference memory task was expressed in terms of latency and distance traveled to find the platform. The animals that did not find the platform within 60 s were gently guided to it.

In the Probe Test, the platform was removed from the pool, and the animals were allowed to swim for 60 s. This test was performed in a single session (on days P25, P39 and P69) 24 h after the end of each reference memory training phase. The percentage of distance traveled and the percentage of time spent within the target quadrant and counter (an area surrounding the platform twice its diameter) [22] were measured to evaluate the spatial bias of the animals within the pool. In addition, the number of crossings in the counter was also recorded. It is important to note that the time spent within the counter reflects a more specific index of the spatial location compared to the time spent within the target quadrant because the counter represents a smaller and more specific location [22].

#### Spatial working memory

Training in the spatial working memory task started 24 h after the probe test. In this task, the platform was placed in a different location every session, one session per day, but was maintained in the same location during the 4 trials within a session. Therefore, in the first trial of each session, the animals were required to find the platform by scanning the pool. Then, on the remaining trials within the same session, the animals were able to use the information acquired in the earlier trials to improve their performance. The spatial working memory was performed in two phases. Phase 1 involved four days (P26 to P29) in infancy and three days in adolescence and adulthood (P40 to P42 and P70 to P72, respectively) (*n* = 12). Phase 2 involved four trials on single days of testing in infancy (P30), adolescence (P43) and adulthood (P73) and were run 24 h after Phase 1 in each case. The intertrial interval (ITI) was 10 minutes in Phase 1, which corresponded to the time required for the animals to maintain the spatial information in working memory, and in Phase 2, the ITI was (virtually) zero minutes, meaning that mice were taken from the platform and immediately began the next trial. The performance on the working memory task was expressed in terms of the distance traveled to the platform scores.

In addition, in the 24-h delay spatial working memory, the percent time spent and the distance traveled within the critical day-before counter was evaluated to provide a 24-h delay test of memory for the precise former platform location (22).

### BDNF

BDNF levels were quantified using the BDNF Emax ImmunoAssay System (Promega, Madison, USA). All samples were analyzed together in a 96-well microplate that was covered with a 100 μl anti-BDNF monoclonal antibody diluted 1:1000 in carbonate buffer (25 mM NaHCO_3_, 25 mM Na_2_CO_3_, pH 9.7). The plate was sealed, incubated overnight at 4°C and washed with Tris-buffered saline containing 0.05% Tween 20. Then, 200 μl/well of 1x Block & Sample buffer were added, and the plate was incubated at room temperature for 1 h. Following additional washes, 100 μl of either the sample (1:4 dilution) or BDNF standard (serial 1:2 dilutions ranging from 0 to 500 pg BDNF/ml) were added, and the plate was incubated for 2 h at room temperature with shaking.

Next, the plate was washed, and 100 μl of the anti-BDNF polyclonal antibody diluted 1:500 in 1x Block & Sample buffer was added. The plate was incubated for 2 h at room temperature with shaking. After additional washes, 100 μl of horseradish peroxidase–conjugated anti-IgY (diluted 1:200 in 1x Block & Sample buffer) was added. The plate was incubated for 1 h at room temperature with shaking and protected from light, and then washed and incubated with 100 μl/well of TMB One solution for 10 minutes with shaking at room temperature. The reaction was stopped by adding 100 μl 1 M HCl, and the absorbance was measured at 450 nm.

### Immunoblotting analysis

The hippocampus was dissected and stored at -80°C until the homogenates were prepared in ice-cold buffer containing 50 mM Tris-HCl (pH 7.4), 1.0 mM PMFS, 10 μg/ml leupeptin, and 1.0 mM L-cit. The extracts were then centrifuged at 1,000 g at 4°C for 5 min. The protein content in the supernatants was measured with the Bradford dye method using the Bio-Rad reagent. The whole extract was treated with Laemmli sample buffer and boiled for 5 min. Equal quantities of protein (synapsin: 10 μg; synaptophysin: 50 μg; PSD95: 50 μg) from each sample were resolved by sodium dodecyl sulfate polyacrylamide gel electrophoresis (SDS-PAGE; 15% polyacrylamide) and transferred onto nitrocellulose membranes. The synaptic proteins of the ETS and control groups were analyzed within each age group (P15, P35 or P65), i.e., no comparisons between ages were performed. After blocking the non-specific sites with 0.2% (w/v) casein, the membranes were incubated overnight at 4–8°C with the following primary antibodies: rabbit polyclonal anti-synapsin, 1:1000 (Millipore, Temecula, United States); mouse monoclonal anti-synaptophysin, 1:1000 (DakoCytomation, Glostrup, Denmark) and mouse monoclonal anti-PSD95 1:1000 (Abcam, Cambridge, United States The same membrane was used for the synapsin and synaptophysin analyses after stripping the first antibody. The membranes were washed with Tris-buffered saline containing 0.1% Tween 20 and then incubated with a peroxidase-conjugated secondary antibody for 1 h. After 3 washes, the immunoreactive bands were visualized using the ECL detection system (Thermo Scientific, Rockford, USA), and the images were captured with ImageQuantTM 400 v.1.0.0 (Amersham Biosciences, Pittsburg, USA). The band intensities were quantified with ImageJ 1.43u (National Institutes of Health, USA), and the results were normalized to the intensity of β-actin (Sigma-Aldrich, St. Louis, USA) [23]. To minimize variability between blots, we evaluated each membrane individually. We quantified and normalized each band using β-actin, and then we averaged the values (protein/β-actin) of all control blots, which were converted to a percentage and expressed as a relative decrease in the protein levels between the test and control samples. The immunoblotting quantification was not blinded and was performed by two observers (LHT and RCTG), who considered the mean of the two observations. S1 File shows the membranes of immunoblotting analysis for synapsin I, synaptophysin and PSD95.

### Immunohistochemistry

The animals were deeply anesthetized (ketamine, 20 mg/100 g of body weight; xylazine, 2 mg/100 g, i.m.) and perfused transcardially with 300 ml of 0.1 M phosphate buffered saline (PBS) followed by 300 ml of 4% paraformaldehyde dissolved in 0.1 M sodium phosphate-buffer (PB), pH 7.4. The brains were then removed and post-fixed for 4 h in the same fixative at 4°C, and then cryoprotected with a 30% sucrose solution (in PB) for 48 h at 4°C. Coronal sections (30 μm) were cut on dry ice using a sliding microtome (Leica SM 2000R —Heidelberger, Nussloch, Germany). The sections were stored in PB at 4°C until use. The sections were stained with rabbit polyclonal anti-synapsin (1:1000) (Millipore, Temecula, USA), and rabbit polyclonal anti-synaptophysin (1:1000) (DakoCytomation, Glostrup, Denmark) primary antibodies that were diluted in PB with 0.3% Triton X-100, and incubated overnight at 22°C. After several PB washes, the brain sections were incubated for 2 h at room temperature with the biotinylated goat anti-rabbit antisera for synapsin and donkey anti-rabbit antisera for synaptophysin (1:200, Jackson ImmunoResearch Lab., West Grove, USA). These antibodies were also diluted in PB containing 0.3% Triton X-100.

Following additional washes (3×10 min), the sections were incubated with the avidin–biotin-peroxidase complex (ABC Elite kit, Vector Labs, Burlingame, USA) for 2 h at room temperature. Labeling was developed with 0.05% diaminobenzidine tetrahydrochloride (DAB) and 0.03% (final concentration) hydrogen peroxide in PB. After the staining procedure, the sections were mounted on glass slides, and the staining was intensified with 0.05% osmium tetroxide in water. The sections were then dehydrated and coverslipped using Permount (Fisher, Pittsburg, USA). The region of interest (CA3 of the hippocampus) was identified using a 20x objective on a Nikon E1000 microscope (Melville, NY, USA). The images were captured using a Nikon DMX1200 digital camera (3–4 sections/animal). To minimize variability among the immunohistochemistry data, we used an optical density ratio. This ratio involves the relationship between the optical density of the region of interest and of the background in each section to obtain an approximate signal-to-noise ratio for each section and for each time point [23]. Immunohistochemistry quantification was performed in the region of interest (CA3), using three randomly selected fields per section from each animal (ImageJ 1.43u—National Institutes of Health, USA). The quantification was not blinded and was performed by two observers (LHT and RCTG), who considered the mean of the two observations.

### Data analyses

The reference memory behavioral data were analyzed using repeated-measures analysis of variance (ANOVA) with the treatments as the between-subject factor and the sessions and trials as the within-subject factors. The probe trial behavioral scores were analyzed using ANOVA with the treatments and sex as the between-subject factors and age as the within-subject factor. *Post-hoc* analysis was performed with the Newman-Keuls test. Separate ANOVAs were performed for each measure.

The means of the distance traveled to the platform for the four trials across the 4 days of testing in infancy and the 3 days of testing in adolescence and adulthood of Phase 1 (10-min ITI) were calculated relative to the working memory behavioral scores. These values, together with the corresponding scores from Phase 2 (0-min ITI), were subjected to an ANOVA with treatment and sex as the between-animal factors and age, trials and ITIs as the within-animal factors. An independent ANOVA was run for each score and each age. *Post-hoc* analysis was performed using the Newman-Keuls test. Separate ANOVAs were performed for each measure.

The BDNF data were analyzed by ANOVA followed by the Newman-Keuls *post hoc* test. Immunoblotting and immunohistochemistry data were analyzed using Student’s t-test for independent samples. The data are presented as the means ± SD or SEM. Differences with a probability of 95% (*p* < 0.05) were considered significant.

## Results

Table 1 shows the levels of CO in the chamber and measurements of the exposure biomarkers: whole-blood COHb, plasma nicotine and cotinine levels.

**Table 1:** CO levels in the chamber (ppm) and exposure biomarkers (whole-blood carboxyhemoglobin and plasma levels of nicotine and cotinine) for control and ETS groups (*n* = 5) immediately after the last exposure. The animals were exposed to ETS from P3 to P14. The data are presented as the means ± SD.

	CO (ppm)	COHb (%)	Nicotine (ng/ml)	Cotinine (ng/ml)
Control (*n* = 5)	not detected	1.5 ± 0.2	not detected	not detected
ETS (*n* = 5)	361.8 ± 77.3	12.5 ± 1.9	126.1 ± 17.2	100.8 ± 14.5

### Spatial reference memory

**Fig 2:**
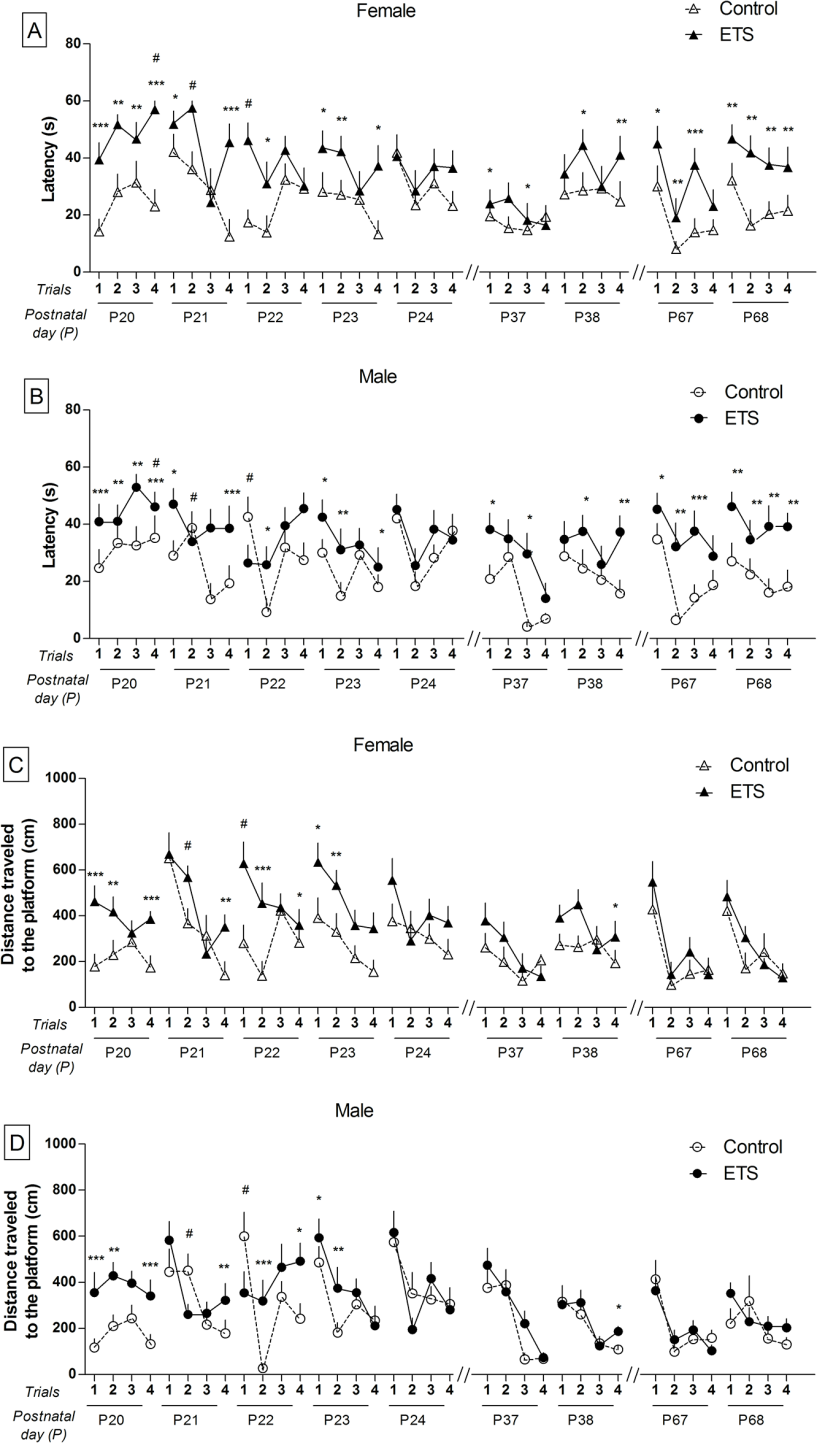
Training in the spatial reference memory task in the Morris water maze (*n* = 12). (A) Latency (females); data expressed in seconds (means ± SEM). (B) Latency (males); data expressed in seconds (means ± SEM). (C) The distance traveled to the platform (females); data expressed in cm (means ± SEM). (D) The distance traveled to the platform (males); data expressed in cm (means ± SEM). Infancy: P20-P24, adolescence: P37-P38, and adulthood: P67-P68. *p < 0.05; **p < 0.01; ***p < 0.001—ETS-exposed mice compared to the control animals. ^#^ *p* < 0.05—treatment x sex interaction.

Fig 2 shows the performance of both the ETS group and the corresponding control animals on the spatial reference memory tests. Exposure to ETS during the early postnatal period disrupted the acquisition of a spatial reference memory task in the water maze, as revealed by the significant treatment effect for latency (Fig 2C and 2D) and distance (Fig 2A and 2B) to the platform in infancy (P20-P24) (F_1,44_ = 53.41–42.14; *p* < 0.0001) and adolescence (P37-P38) (F_1,44_ = 12.93–8.97; *p* < 0.01). In adulthood (P67-P68), there was a significant treatment effect for latency (F_1,44_ = 47.70; *p* < 0.0001), but not for distance to the platform (F_1,44_ = 2.00; *p* > 0.05). In addition, we observed a session effect for latency and distance to the platform in infancy (F_4,176_ = 3.21–4.04; *p* < 0.05 = 0.0143) and distance to the platform in adolescence (F_1,44_ = 11.51; *p* < 0.01); and a trial effect for latency and distance to the platform in infancy (F_3,132_ = 5.05–22.72; *p* < 0.01), adolescence (F_3,132_ = 6.45–20.21; *p* < 0.001) and adulthood (F_3,132_ = 19.86–30.95; *p <* 0.0001). **S1 Table** shows a detailed description of the statistical analysis. The results show both poorer acquisition and performance by the ETS animals relative to controls and a greater susceptibility of females to ETS exposure compared to males, particularly in infancy.

**Fig 3:**
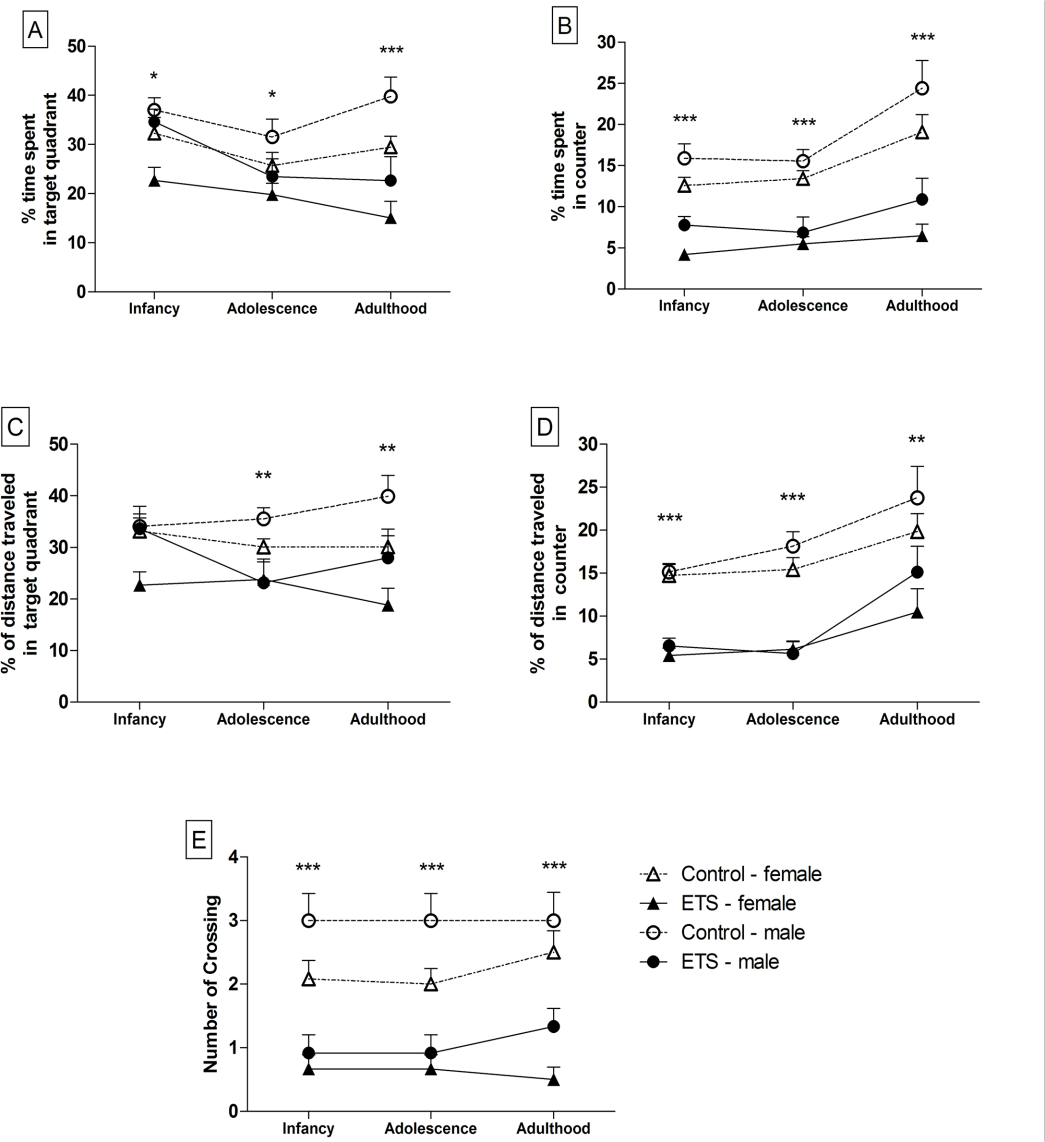
Probe Test following the spatial reference memory training in the Morris water maze (*n* = 12). Infancy (P25), adolescence (P39) and adulthood (P69). (A) Time spent within the target quadrant; the data are expressed as the percentage of time in the target quadrant (s) relative to the total time (s) in the water maze (means ± SEM). (B) Time within the counter; the data are expressed as the percentage of time in the counter (s) relative to the total time (s) in the water maze (means ± SEM). (C) Distance traveled within the target quadrant; the data are expressed as the percentage of the distance traveled in the target quadrant (cm) relative to the total distance traveled (cm) in the water maze (means ± SEM). (D) Distance traveled within the counter; the data are expressed as the percentage of distance traveled within the counter (cm) relative to the total distance traveled (cm) within the water maze (means ± SEM). (E) Number of crossings in the original location of the platform; the data are expressed in absolute numbers (means ± SEM). *p < 0.05; **p < 0.01; ***p < 0.001—ETS-exposed mice compared with the control animals.

During the Probe Test, the animals that were exposed to ETS in the early postnatal period exhibited a disruption in recall of the platform location when the platform was removed (Fig 3). The ANOVA revealed significant treatment effects for both time and distance traveled in the target quadrant (F_1,44_ = 25.07–16.68; *p* < 0.001) and in the counter (F_1,44_ = 73.86–69.15; *p* < 0.0001), with these values being significantly lower in the animals exposed to ETS than in the controls (Fig 3A to 3D). This observation was confirmed by the substantial reduction in the number of times the ETS animals crossed the area corresponding to the original location of the platform (F_1,44_ = 55.53; *p* < 0.0001) (Fig 3E).

The results also show a significant sex effect, as detailed in S1 Table, indicating that female mice performed more poorly than male animals. In addition, the ANOVA revealed a significant age effect in the time spent within the target quadrant (F_2,88_ = 4.26; *p* < 0.05) and in both time spent (F_2,88_ = 12.31; *p* < 0.0001) and distance traveled (F_2,88_ = 14.81; *p* < 0.0001) within the counter. Further analysis revealed that control adult mice showed an increase in the time spent (F_2,69_ = 9.171; *p* < 0.001) and distance traveled (F_2,69_ = 6.050; *p* < 0.01) within the counter compared to the values obtained in their infancy and adolescence (Fig 3B and 3D). However, adult mice exposed to ETS showed a decrease in the time spent within the target quadrant compared to their infancy (F_2,69_ = 9.171; *p* < 0.05), and an increase in the distance traveled (F_2,69_ = 9.028; *p* < 0.001) within the counter compared to the values obtained in their infancy and adolescence (Fig 3A and 3D).

### Spatial working memory

**Fig 4:**
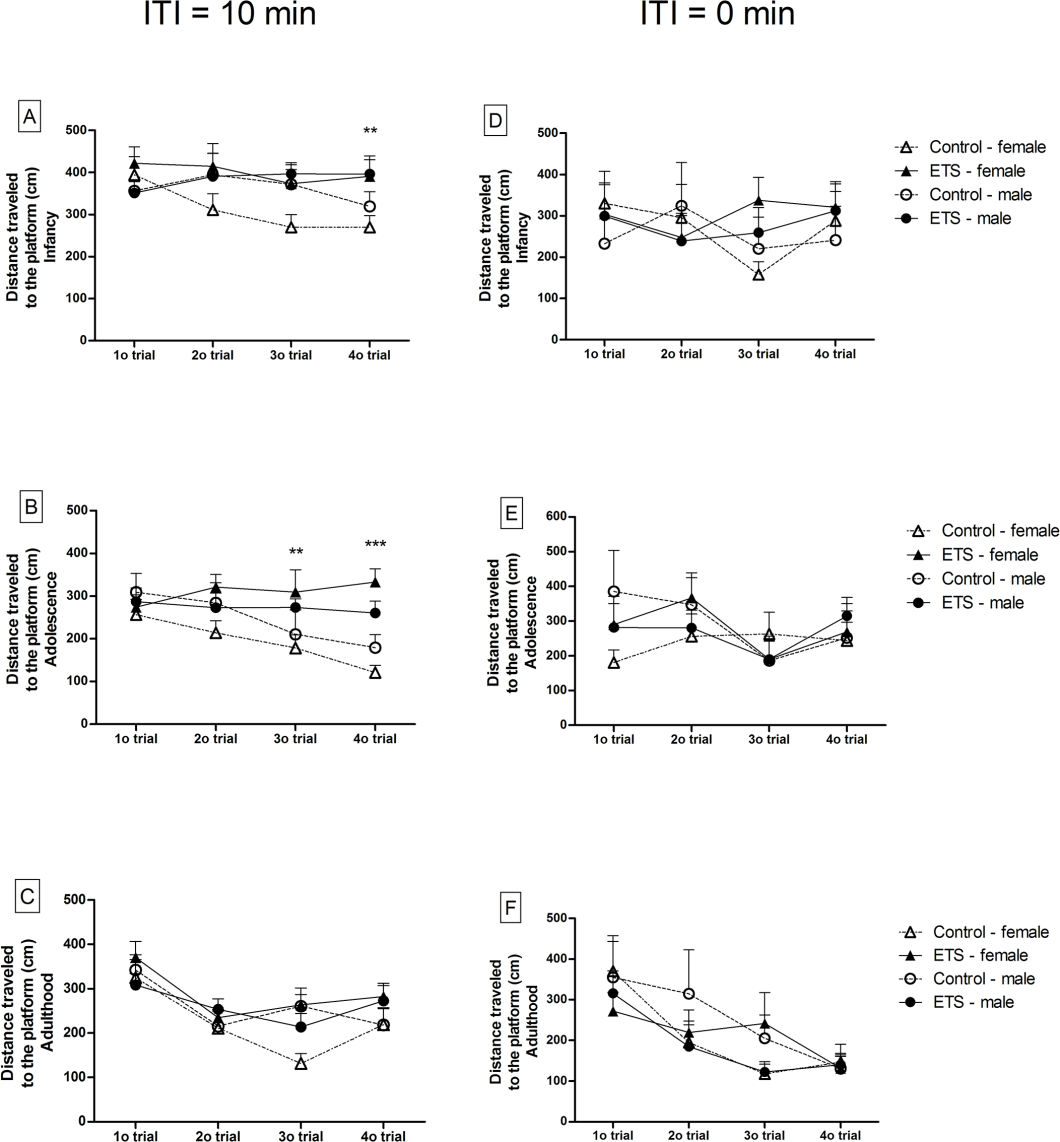
Distance traveled to the platform in a spatial working memory task (*n* = 12). The data are expressed in cm (means ± SEM). A-F: Phase 1 (10-min ITI). A-C: Females; D-F: Males. A/D—infancy (P26-P29), B/E—adolescence (P40-P42) and C/F—adulthood (P70-P72). Trials 1–4 were averaged over the 4 days of testing in infancy and the 3 days of testing in adolescence and adulthood. G-L: Phase 2 (0-min ITI). G/J—infancy (P30), H/K—adolescence (P43) and I/L—adulthood (P73). **p < 0.01; ***p < 0.001—ETS-exposed mice compared with the control animals.

The water-maze spatial working memory task performance was evaluated using the distance traveled to the platform. Phase 1 (Fig 4A–4F), involving 10 min of ITI, was performed during 4 days in infancy (P26-P29) and 3 days in adolescence (P40-P42) and adulthood (P70-P72). The mice that were exposed to ETS showed a deficit in the spatial working memory during these trials, which was suggestive of a deficit in either acquisition or maintenance of the critical information regarding the platform location. Therefore, the ITI was reduced to 0 min (Phase 2) to assess these possibilities (Fig 4G–4L). S1 Table shows the statistical effects regarding spatial working memory.

As shown in Fig 4, the distance to the platform from trial 1 to 4 quickly improved for control mice in Phase 1 during infancy and adolescence (Fig 4A, 4B, 4D and 4E), whereas the mice exposed to ETS did not show improvements at these ages. In contrast, in Phase 2, there were no differences between the ETS and control groups (Fig 4G–4L). Phase 2 (0-min ITI) indicates that the animals do not show deficits in sensory perception or motor performance because they are immediately exposed to the maze at the end of each trial. Therefore, these results discard the possibility that the deficits in memory performance observed in Phase 1 are due to sensory or motor deficits.

In adulthood, there were no differences between the ETS and control groups in either Phase 1 or 2 (Fig 4C, 4F, 4I and 4L). There was a significant decrease in the distance to the platform in trials 2–4 compared to the first trial in both Phases 1 (F_3,188_ = 10.56; *p* < 0.0001) and 2 (F_3,188_ = 8.01; *p* < 0.0001), indicating that the effects of ETS were reversed at this age. In addition, given the significant treatment x sex interaction for the distance to the platform (S1 Table), the results confirm that the females are more susceptible to the ETS exposure than the males.

**Fig 5:**
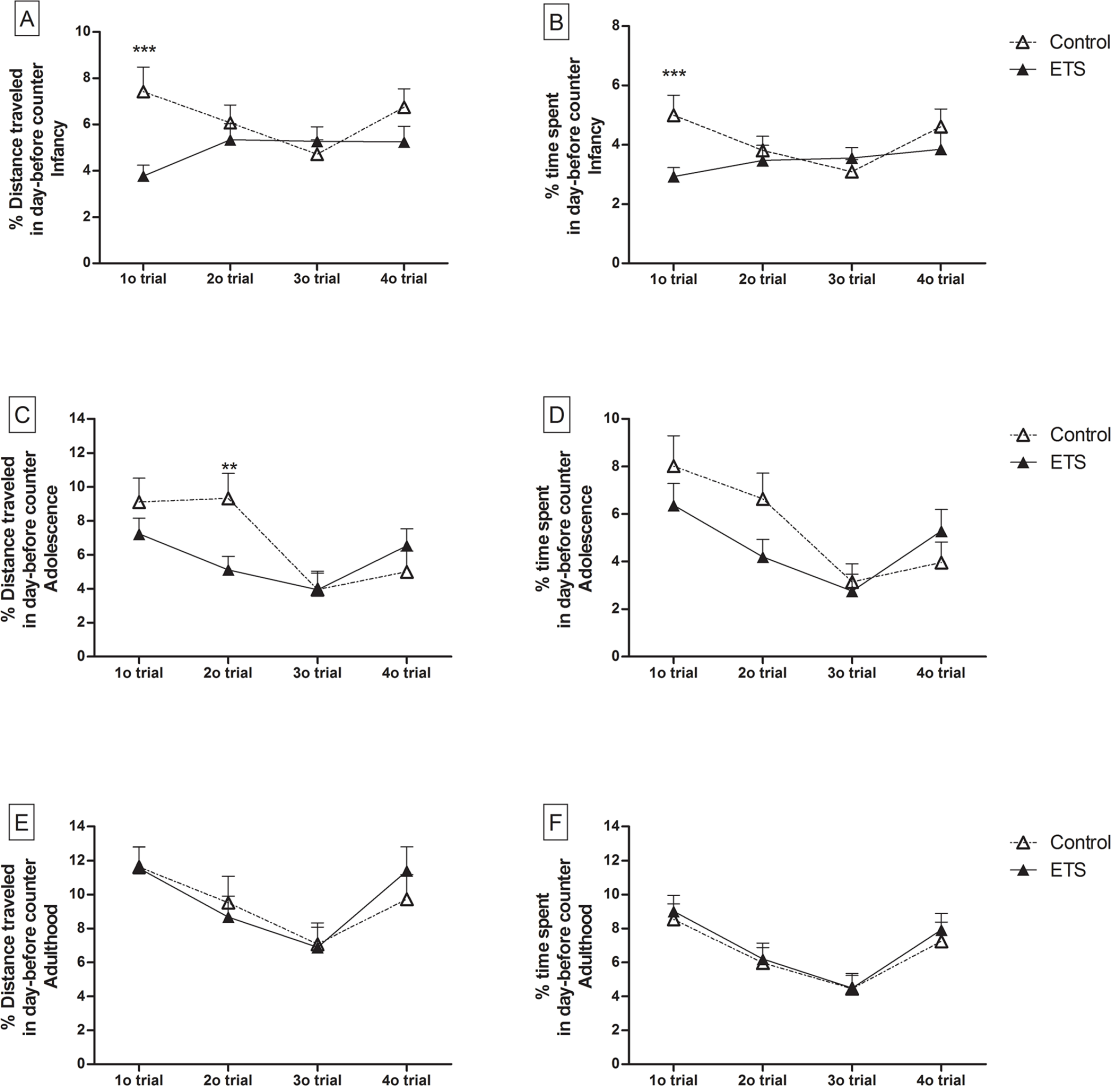
Distance traveled and time spent in the day-before counter in the spatial working memory task. The data are expressed as the percentage of the distance traveled (cm) or time spent (s) in the day-before counter relative to the total distance traveled (cm) or to the total time spent (s) in the water maze (*n* = 24) (means ± SEM). A-B: infancy (P26-P29), C-D: adolescence (P40-P42) and E-F: adulthood (P70-P72). Trials 1–4 were averaged over the 4 days of testing in infancy and the 3 days of testing in adolescence and adulthood. **p < 0.01; ***p < 0.001—ETS-exposed mice compared with the control animals.

The distance traveled and time spent in the counter where the platform was located on the previous day (day-before counter) were analyzed to evaluate the 24-h delayed working memory (Fig 5). We observed a significant treatment effect for the distance traveled (F_1,46_ = 9.99; *p* < 0.01) (Fig 5A) and the time spent (F_1,46_ = 7.81; *p* < 0.01) (Fig 5D) in the day-before counter on the first trial in infancy. In addition, a significant treatment effect was observed in the distance traveled (F_1,46_ = 6.27; *p* < 0.05) (Fig 5B) in the day-before counter on the second trial in adolescence. These results indicate that mice exposed to ETS in the early postnatal period have impairments in 24-h delayed working memory at these ages. There were no differences in the day-before counter during adulthood.

Furthermore, there were significant age and trial effects, as well as a significant age x trial interaction, for both the time and distance traveled in the day-before counter. There were no significant differences between sexes (S1 Table).

### BDNF

**Fig 6:**
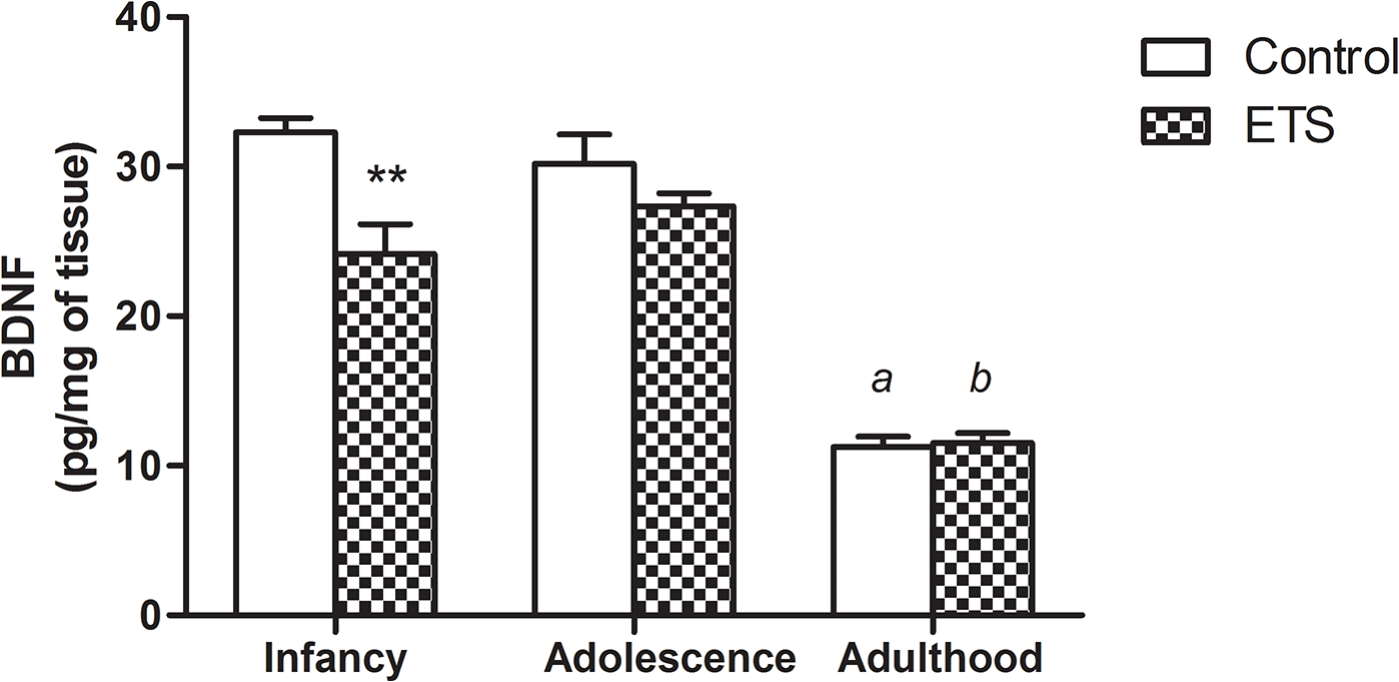
BDNF levels in the hippocampus using BDNF Emax ImmunoAssay System kit (*n* = 6). The data are expressed in pg*mg^-1^ of tissue (means ± SEM). **p < 0.01 –infant mice exposed to ETS in the early postnatal period compared to the control animals of the same age. a—*p* < 0.001 –adult control *vs*. infancy and adolescence controls. b—*p* < 0.001 –adult ETS-exposed mice *vs*. infancy and adolescent ETS-exposed mice.

A two-way ANOVA revealed a significant treatment (F_1,30_ = 11.04; *p* < 0.01) and age (F_2,30_ = 112.3; *p* < 0.0001) effect, as well a significant treatment x age interaction (F_2,30_ = 5.158; *p* < 0.05). As shown in Fig 6, exposure to ETS in the early postnatal period induced a significant decrease in BDNF levels in infancy compared to the controls; no differences were observed between the ETS-exposed mice and control animals in either adolescence or adulthood. However, there was a significant decrease in the BDNF levels in both the ETS-exposed and unexposed adult mice compared with the observed levels in infancy and adolescence.

### Immunohistochemistry and Immunoblotting

**Fig 7:**
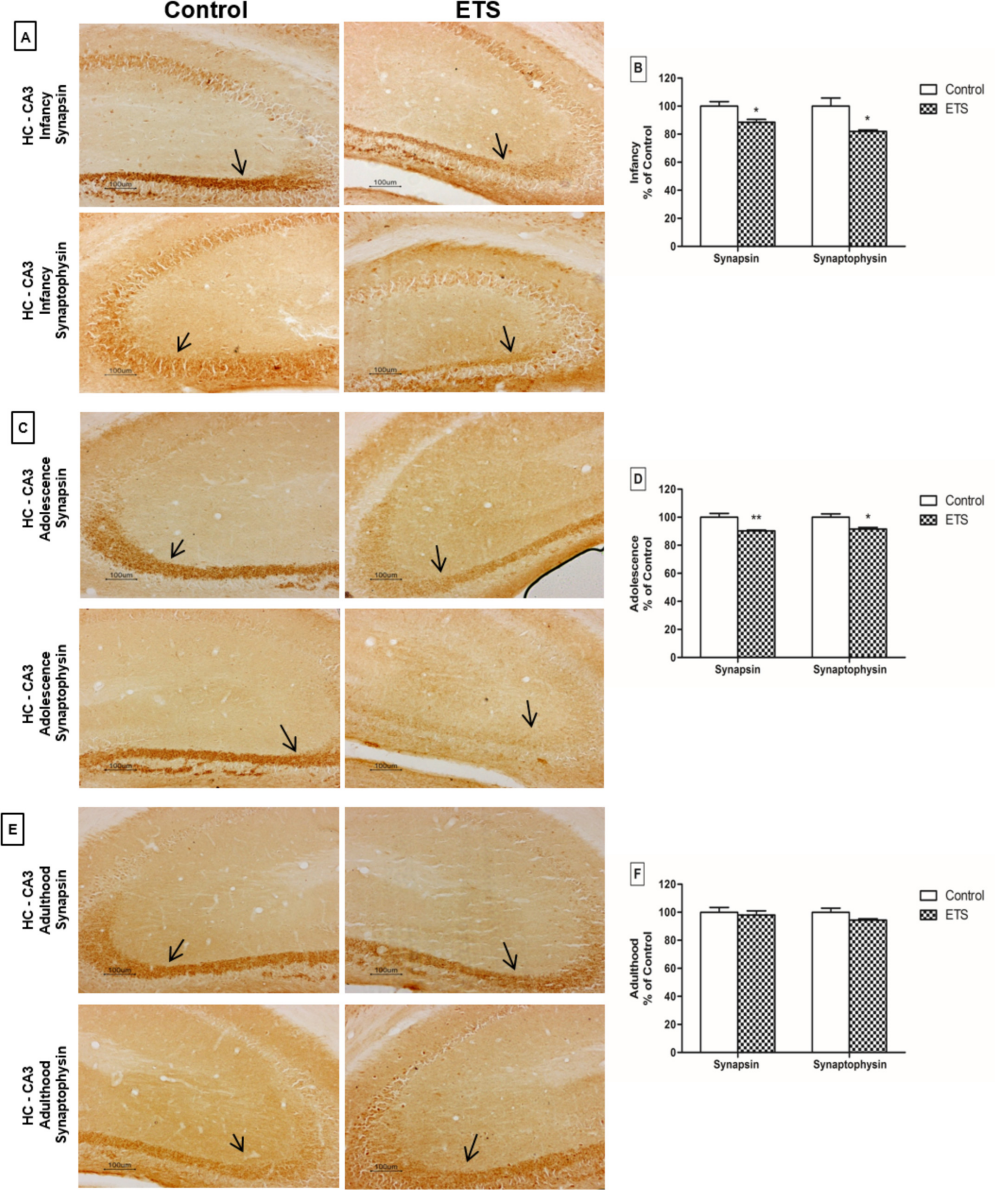
Effects of ETS exposure during the early postnatal period on synaptic proteins by immunohistochemistry. Synapsin I and synaptophysin in the hippocampus in infancy (A-B), adolescence (C-D) and adulthood (E-F). A, C and E—Digital images of coronal sections of the CA3 region stained for synapsin I and synaptophysin. B, D and F—Quantification of synapsin I and synaptophysin levels by averaging the ratio of the optical density in the CA3 region and background in each section. The quantification was performed using three randomly selected fields per section of each animal (*n* = 5 animals). The data are expressed as the percent of control (means ± SEM). The percent of the control was calculated using arbitrary units. * *p* <0.05; **p < 0.01 –mice exposed to ETS in the early postnatal period compared to the controls. Arrows: synapsin I or synaptophysin immunoreactivity in the CA3 region of the hippocampus. Scale bar: 100 μm.

**Fig 8:**
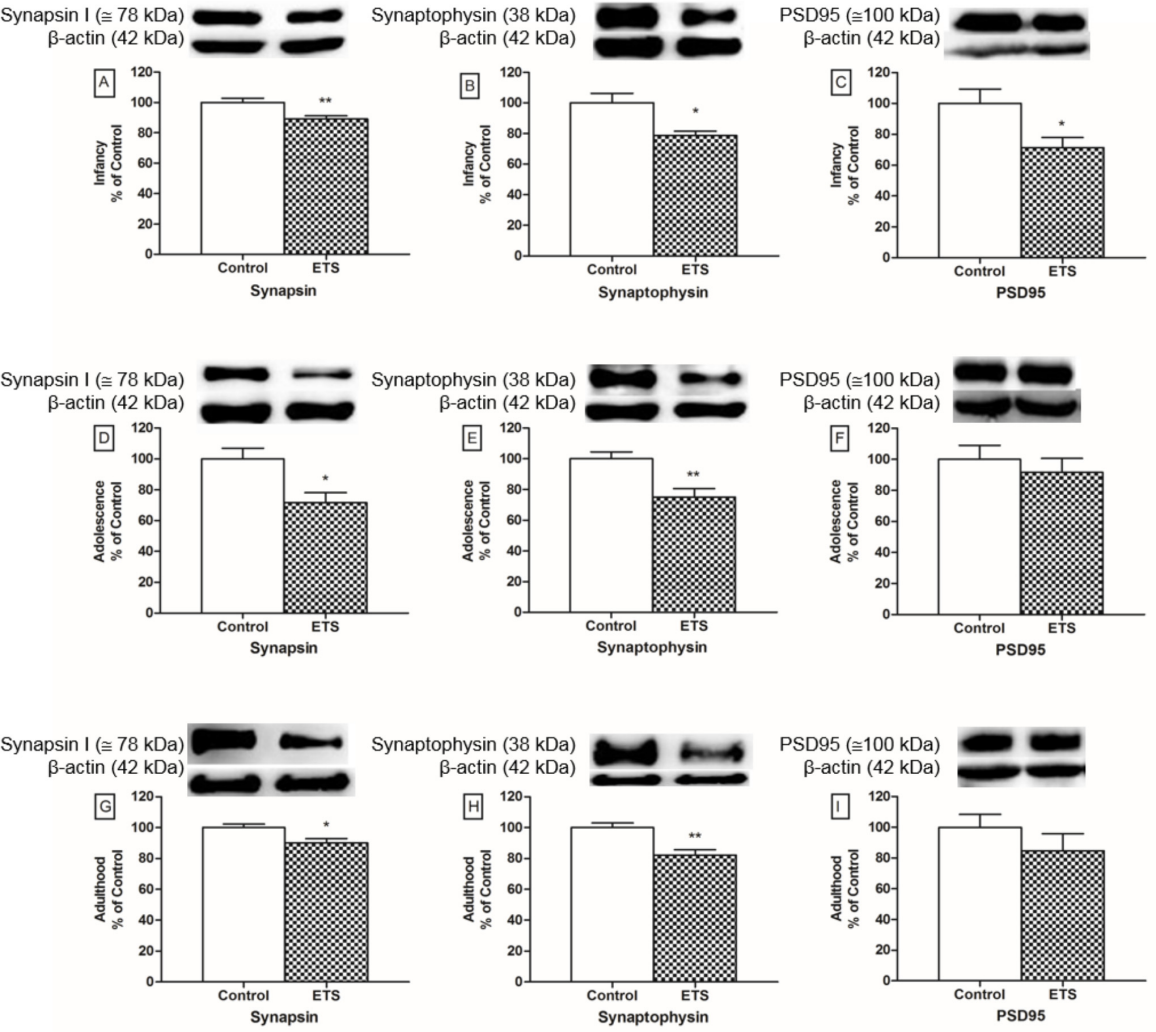
Effects of ETS exposure during the early postnatal period on synaptic proteins by immunoblotting. Synapsin I, synaptophysin and PSD95 levels in the hippocampus (n = 6 animals) in infancy (A-C), adolescence (D-F) and adulthood (G-I) of mice exposed to ETS in the early postnatal period compared to the controls in infancy. The data are expressed as the percent of the control (means ± SEM). The percent of the control was calculated using arbitrary units. *p < 0.05; **p < 0.01. Synapsin I (≅ 78 kDa), Synaptophysin (38 kDa), PSD95 (≅ 100 kDa), β-actin (42 kDa).

The immunohistochemistry analyses showed that the exposure to ETS during the early postnatal period induced significant decreases in the immunoreactivity of synapsin I and synaptophysin (*p* < 0.05 for each, t-test) in the hippocampi of infant mice (Fig 7A and 7B). These findings were confirmed by immunoblotting (Fig 8A and 8B), which also showed decreases in the synapsin I and synaptophysin protein levels (*p* < 0.01 and *p* < 0.05, respectively; t-test). Furthermore, we observed that ETS exposure induced a decrease in the PSD95 protein level in the hippocampus of infant mice (*p* < 0.05, t-test), as shown in Fig 8C.

The effects of ETS exposure in the early postnatal period on the pre-synaptic proteins persisted into adolescence. Decreases in both synapsin I and synaptophysin immunoreactivity (*p* < 0.01 and *p* < 0.05, respectively; t-test) (Fig 7C and 7D) and protein levels (*p* < 0.05 for both, t-test, Fig 8D and 8E) were observed. However, no difference was observed for PSD95 during adolescence (Fig 8F).

In adult mice, ETS exposure during the early postnatal period induced decreases in both the synapsin I and synaptophysin protein levels (*p* < 0.05 and *p* < 0.01, respectively; t-test, Fig 8G and 8H). No differences were observed in synapsin I and synaptophysin immunoreactivity or in PSD95 protein levels during adulthood (Figs 7E, 7F and 8I).

S1 File shows the membranes of immunoblotting analysis for synapsin I, synaptophysin and PSD95.

## Discussion

To the best of our knowledge, this is the first study showing the effects of ETS exposure during the early postnatal period on infant mouse brain development and the consequences of these effects in adolescence and adulthood. The current findings indicate that exposure to ETS during the early postnatal period induces impairments in learning and memory, which can be related to reduced levels of both synaptic proteins and BDNF.

It was critical to use the same animals from infancy to early adulthood for the behavioral tests, given that our aim was to assess the effects of the exposure to ETS in the early postnatal phase and its impact in later phases of development. One possible criticism of using the same set of animals throughout the study is that any alteration observed in adolescence or in adulthood, could be interpreted as a response to the exposure to ETS in the early postnatal period or as a consequence of repeated experience in the behavioral trials. In fact, the goal of this study was to evaluate the long-term consequences of ETS in the postnatal period on memory and synaptic proteins. As we observed a deleterious effect in infancy, adolescence and in adulthood, our results reinforce the idea that passive smoke induced an impairment during a critical period of brain development.

The Morris water maze results suggest that the exposure to ETS during the early postnatal period disrupted spatial reference memory at all evaluated ages. A recent study evaluated the effects of exposure to tobacco smoke during gestation and the early postnatal period on cognitive functions in adult mice [24]. Compared to the controls, the animals exposed to tobacco smoke exhibited poorer performance in (i) the last day of training in a visible-platform version of the Morris water maze task and (ii) the hidden-platform version of the reference memory test in the Morris water maze. In contrast, the animals exposed to the smoke showed no disturbances during the performance of a matching-to-sample Morris water maze (a type of spatial working memory task) [24]. Moreover, another study evaluated the correlation between cotinine urine levels, attention deficit hyperactivity disorder (ADHD) and learning disability symptoms in children aged approximately 9 years. The authors found that children exposed to ETS had a higher risk of ADHD and learning disabilities [25].

The ETS exposure not only impaired spatial reference memory but also working memory, as evidenced by the 10-minute intertrial interval task in infancy and adolescence and in the 24-h delay spatial search. In humans, working memory refers to complex cognitive tasks, such as reasoning, language comprehension and learning, which involve the temporary storage and processing of information [26]. Consistent with our results, studies have reported that prenatal tobacco smoke is associated with impaired performance on working memory tasks in 4-year-old children and in adolescents [27,28].

Our results suggest that females are more susceptible to the effects of ETS on learning and memory than males. In fact, a clinical study found that adolescent smokers with prenatal exposure to maternal smoking showed impaired auditory and visual attention, with girls presenting a decrease in both attentional functions and boys only in auditory attention [29]. In rodents, electrophysiological studies revealed that males responded higher than females to nicotinic receptor stimulation in the prefrontal attention circuitry during the first postnatal month [19]. Nicotinic receptors induce the maturation of the corticothalamic circuitry during development [30], which may contribute to sex differences in attention-related behaviors [19]. In addition, the administration of nicotine to male adolescent (P30-P47) rats demonstrated an increase in serotonin turnover at 13 and 23 days of withdrawal of a nicotine challenge during adulthood (P90-P107). In contrast, females showed a decrease in serotonin turnover 3 days after the nicotine challenge [20]. Together, these data indicate gender differences in response to tobacco or nicotine.

The hippocampus is a critical structure that is responsible for spatial learning and memory, processes that are dependent on neural plasticity and changes in the connectivity between neurons. Neurotrophins, such as BDNF, are required for memory consolidation and they may regulate hippocampal neurogenesis and synaptogenesis, which are essential for learning and memory [31–33]. Several studies have shown that BDNF can modulate the expression of pre- and postsynaptic proteins. Mice lacking synapsin I and/or synapsin II showed robust decreases in BDNF-mediated glutamate release, suggesting a link between synapsin phosphorylation and BDNF [34]. Furthermore, BDNF knockout mice exhibit impairments in neurotransmitter release, as revealed by the decreases in the number of docked vesicles and in the levels of synaptic proteins (synaptophysin and synaptobrevin) [35]. Consistent with these observations, the expression of the TrkB receptors, which can be activated by BDNF, and synapsin I were increased in the hippocampus of rats submitted to a spatial memory task in the Morris water maze [36]. Moreover, BDNF promoted synaptogenesis by enhancing synapsin and PSD95 expression [37].

The ETS-induced decrease in BDNF levels in infancy is consistent with the reductions in the synapsin, synaptophysin and PSD95 protein levels, as well as the learning and memory impairments observed at this age. The importance of BDNF in brain development was emphasized by the reduction of this neurotrophin during adulthood in both the control and ETS-exposed mice. Synaptophysin is related to the presynaptic terminal [38,39]. Before postnatal day 10, the presynaptic short-term plasticity and the number of synaptic vesicles are independent of synapsin. In contrast, beginning at postnatal day 20, the function and maturation of excitatory synapses are closely linked to on synapsin [40].

It is noteworthy that synapsin and synaptophysin were decreased in all ages, as shown by immunoblotting analysis. However, the immunohistochemistry results do not match the immunoblotting results in adulthood, suggesting that the differences may result from other areas than CA3 that may be involved in the ETS-induced reductions of the synaptic proteins.

Studies with synapsin knockout mice demonstrated deficits in learning and memory, showing that synapsins are essential for the maintenance of the number of synaptic vesicles by accelerating vesicle trafficking during repetitive stimulation and regulating short-term synaptic plasticity. However, synapsins are not required for synaptogenesis, neurite outgrowth, or synaptic vesicle trafficking [41–43]. PSD95 expression is associated with the maturation of glutamatergic synapses in the hippocampus via interactions with both AMPARs and NMDA-type glutamate receptors (NMDARs). PSD95 increases the number and the size of dendritic spines and regulates NMDAR-dependent changes in the number of AMPARs, suggesting that PSD95 contributes to synapse stabilization and plasticity [44,45]. Several studies associate alterations in memory with synaptic proteins. Environmental enrichment can be a potent cognitive enhancer for aged female mice, and showed a positive correlation between increased synaptophysin and memory [46]. However, synaptophysin knockout mice showed reduced object novelty recognition and impairments in learning and memory [47]. Moreover, the expression of different synapsin isoforms in the hippocampus is correlated with the improvements in spatial memory induced by SGS742, a cognitive enhancer. These previous results strengthen the importance of these synaptic proteins in the memory process [48].

The nicotine, cotinine and COHb levels observed in the present study are consistent with those reported in previous studies in rodents [9,10,24,49]. There is an important difference between the COHb levels in humans and mice. While COHb remains constant throughout the day in humans due to a long half-life of approximately 4–5 hs, COHb has a half-life of 30 minutes in mice due the labile bond between the carbon monoxide and hemoglobin [50]. In children exposed to ETS, the nicotine and cotinine urine levels are consistent with the number of smokers with whom they are in contact and the extent of the exposure. A study with hospitalized children showed that out of 72 urine samples, 17 were positive for cotinine and three for nicotine, with values between 6.90–272.60 ng/mL and 13.55–188.40 ng/mL, respectively [51]. Although it is not possible to directly correlate ETS exposure in humans and rodents, studies with this focus can contribute to the planning of public policies to prevent the damage caused by passive smoke.

The present study combined biochemical and behavioral data to assess the effects of the exposure to ETS from infancy to early adulthood. In summary, mice exposed to ETS during early postnatal development exhibit impairments in spatial reference and working memory, and these results can be related to decreases in the hippocampal levels of synaptic proteins and BDNF. Thus, the present study shows that ETS leads to changes in mouse brain development and disrupts cognition functions, with most of these effects being permanent.

## Supporting Information

S1 Table Detailed description of the statistical analysis of the Morris Water Maze data. *p < 0.05; **p < 0.01; ***p < 0.001; CO = counter; TQ = target quadrant.
(DOCX)

S1 File Membranes of immunoblotting analysis for synapsin I, synaptophysin and PSD95. A/B—synapsin I in infant mice; C/D—synapsin I in adolescent mice; E/F—synapsin I in adult mice; G/H—synaptophysin in infant mice; I/J—synaptophysin in adolescent mice; K/L—synaptophysin in adult mice; M/N–PSD95 in infant mice; O/P—PSD95 in adolescent mice; Q/R—PSD95 in adult mice.
(ZIP)
