# Supplementary material for: Exposure of Neonatal Mice to Tobacco Smoke Disturbs Synaptic Proteins and Spatial Learning and Memory from Late Infancy to Early Adulthood
Source: PLoS One. 2015 Aug 25;10(8):e0136399. doi: 10.1371/journal.pone.0136399 (PMC4549279; doi:10.1371/journal.pone.0136399)
Supplement: S1 Table — *p < 0.05; **p < 0.01; ***p < 0.001; CO = counter; TQ = target quadrant. (DOCX) [file pone.0136399.s001.docx]

Table S1: Detailed description of the statistical analysis in Morris Water Maze. **p* < 0.05; ***p* < 0.01; ****p* < 0.001; CO = Counter; TQ = target quadrant.

| **Statistical effect** | | **F** | | | |
| --- | --- | --- | --- | --- | --- |
| ***Spatial reference memory*** | |  | |  |  |
| ***Training*** | | Infancy | | Adolescence | Adulthood |
| Treatment effect | Latency | F_1,44_ = 53.41; *p* < 0.001*** | | F_1,44_ = 12.93; *p* < 0.01** | F_1,44_ = 47.70; *p* < 0.001*** |
|  | Distance traveled to the platform | F_1,44_ = 42.14; *p* < 0.001*** | | F_1,44_ = 8.97; *p* < 0.01** | - |
| Session effects | Latency | F_4,176_ = 3.21; *p* < 0.01* | | - | - |
|  | Distance traveled to the platform | F_4,176_ = 4.04; *p* < 0.01* | | F_1,44_ = 11.51; *p* < 0.01** | - |
| Trial effects | Latency | F_3,132_ = 5.05; *p* < 0.01* | | F_3,132_ = 6.45; *p* < 0.001*** | F_3,132_ = 19.86; *p* < 0.001*** |
|  | Distance traveled to the platform | F_3,132_ = 22.72; *p* < 0.01* | | F_3,132_ = 20.21; *p* < 0.001*** | F_3,132_ = 30.95; *p* < 0.001*** |
| Treatment x Session interaction | Distance traveled to the platform | F_4,176_ = 3.55; *p* < 0.01* | | - | - |
| Session x Trial interaction | Latency | F_12,528_ = 6.49; *p* < 0.001*** | | - | F_3,132_ = 3.09; *p* < 0.001*** |
|  | Distance traveled to the platform | F_12,528_ = 5.36; *p* < 0.001*** | | - | F_3,132_ = 5.36; *p* < 0.001*** |
| Session x Sex interaction | Distance traveled to the platform | - | | F_1,44_ = 7.30; *p* < 0.01** | - |
| Trial x Sex interaction | Latency | F_3,132_ = 2.84; *p* < 0.05* | | - | - |
|  | Distance traveled to the platform | - | | F_3,132_ = 2.73; *p* < 0.05* | F_3,132_ = 2.71; *p* < 0.05* |
| Trial x Treatment x Sex interaction | Latency | F_3,132_ = 2.99; *p* < 0.05* | | - | - |
| Session x Trial x Treatment interaction | Latency | - | | - | F_3,132_ = 3.67; *p* < 0.05* |
| Treatment x Sex x Session x Trial interaction | Latency | F_12,528_ = 2.62; *p* < 0.01** | | - | - |
|  | Distance traveled to the platform | F_12,528_ = 2.05; *p* < 0.05* | | - | - |
| ***Probe*** | |  | | | |
| Treatment effects | % of time in the TQ | F_1,44_ = 25.07; *p* < 0.001*** | | | |
|  | % distance traveled in the TQ | F_1,44_ = 16.68; *p* < 0.001*** | | | |
|  | % of time in the CO | F_1,44_ = 73.86; *p* < 0.001*** | | | |
|  | % distance traveled in the CO | F_1,44_ = 69.15; *p* < 0.001*** | | | |
|  | Number of crossing - platform area | F_1,44_ = 55.53; *p* < 0.001*** | | | |
| Sex effect | % of time in the CO | F_1,44_ = 8.55; *p* < 0.01** | | | |
|  | % of time in the TQ | F_1,44_ = 14.81; *p* < 0.001*** | | | |
|  | % distance traveled in the TQ | F_1,44_ = 7.62; *p* < 0.01** | | | |
|  | Number of crossing - platform area | F_1,44_ = 6.97; *p* < 0.05* | | | |
| Age effect | % of time in the TQ | F_2,88_ = 4.26; *p* < 0.05* | | | |
|  | % of time in the CO | F_2,88_ = 12.31; *p* < 0.001*** | | | |
|  | % distance traveled in the CO | F_2,88_ = 14.81; *p* < 0.001*** | | | |
| *Adult vs infancy and adolescence – Control group* | % of time in the CO | F_2,69_ = 9.171; *p* < 0.001*** | | | |
|  | % distance traveled in the CO | F_2,69_ = 6.050; *p* < 0.01** | | | |
| *Adult vs infancy – ETS group* | % of time in the TQ | F_2,69_ = 9.171; *p* < 0.05* | | | |
| *Adult vs infancy and adolescence – ETS group* | % distance traveled in the CO | F_2,69_ = 9.028; *p* < 0.001*** | | | |
| ***Spatial working memory*** | |  | |  |  |
| Treatment effect | Distance traveled to the platform | | F_1,44_ = 4.17; *p* < 0.05* | | |
| Phase effect | Distance traveled to the platform | | F_1,44_ = 14.45; *p* < 0.001*** | | |
| Trial effect | Distance traveled to the platform | | F_3,132_ = 11.48; *p* < 0.001*** | | |
| Treatment x Sex interaction | Distance traveled to the platform | | F_1,44_ = 4.62; *p* < 0.05* | | |
| Phase x Treatment interaction | Distance traveled to the platform | | F_1,44_ = 6.12; *p* < 0.05* | | |
| Trial x Treatment interaction | Distance traveled to the platform | | F_3,132_ = 2.38; *p* < 0.05* | | |
| Age x Phase interaction | Distance traveled to the platform | | F_2,88_ = 4.84; *p* < 0.05* | | |
| Age x Trial interaction | Distance traveled to the platform | | F_6,264_ = 3.14; *p* < 0.01** | | |
| 1^st^ trial vs 2^nd^, 3^rd^ and 4^th^ trials |  | |  | | |
| Phase 1 | Distance traveled to the platform | | F_3,188_ = 10.56; *p* < 0.001*** | | |
| Phase 2 | Distance traveled to the platform | | F_3,188_ = 8.01; *p* < 0.001*** | | |
| **Day-before counter** |  | |  | | |
| Treatment effects - 1^st^ trial in childhood | % of time in the day-before CO | | F_1,46_ = 7.81; *p* < 0.01** | | |
|  | % distance traveled in the day-before CO | | F_1,46_ = 9.99; *p* < 0.01** | | |
| Treatment effects - 2^nd^ trial in adolescence | % distance traveled in the day-before CO | | F_1,46_ = 6.27; *p* < 0.05* | | |
| Age effect | % of time in the day-before CO | | F_2,88_ = 21.21; *p* < 0.001*** | | |
|  | % distance traveled in the day-before CO | | F_2,88_ = 16.95; *p* < 0.001*** | | |
| Trial effect | % of time in the day-before CO | | F_3,132_ = 8.92; *p* < 0.001*** | | |
|  | % distance traveled in the day-before CO | | F_3,132_ = 15.40; *p* < 0.001*** | | |
| Age x Trial interaction | % of time in the day-before CO | | F_6,264_ = 2.38; *p* < 0.05* | | |
|  | % distance traveled in the day-before CO | | F_6,264_ = 3.65; *p* < 0.05* | | |
