# Supplementary figures and images for: Exposure of Neonatal Mice to Tobacco Smoke Disturbs Synaptic Proteins and Spatial Learning and Memory from Late Infancy to Early Adulthood
Source: PLoS One. 2015 Aug 25;10(8):e0136399. doi: 10.1371/journal.pone.0136399 (PMC4549279; doi:10.1371/journal.pone.0136399)

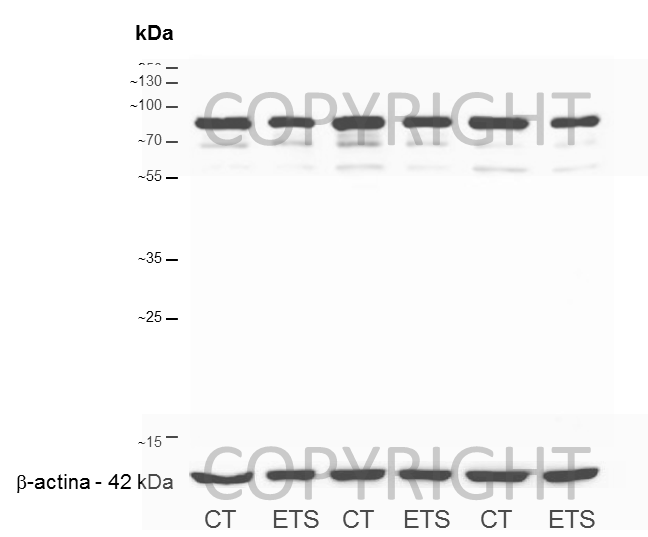

Supplement: S1 File — A/B—synapsin I in infant mice; C/D—synapsin I in adolescent mice; E/F—synapsin I in adult mice; G/H—synaptophysin in infant mice; I/J—synaptophysin in adolescent mice; K/L—synaptophysin in adult mice; M/N–PSD95 in infant mice; O/P—PSD95 in adolescent mice; Q/R—PSD95 in adult mice. (ZIP) [file pone.0136399.s002.zip › Figure S/Figure S1A - SYN 15d m1.tif]

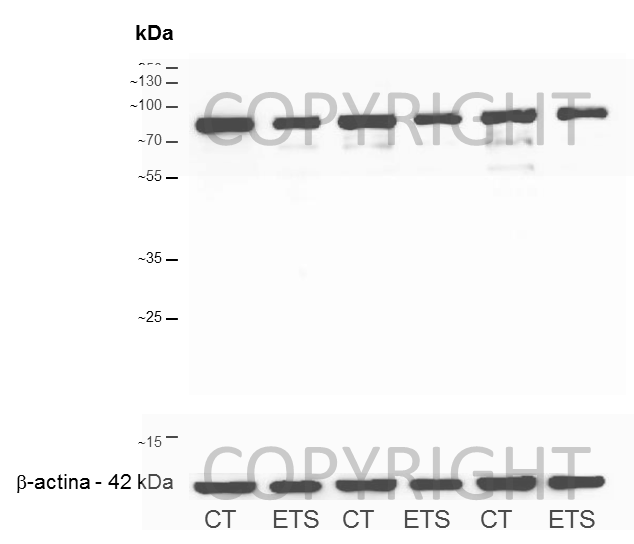

Supplement: S1 File — A/B—synapsin I in infant mice; C/D—synapsin I in adolescent mice; E/F—synapsin I in adult mice; G/H—synaptophysin in infant mice; I/J—synaptophysin in adolescent mice; K/L—synaptophysin in adult mice; M/N–PSD95 in infant mice; O/P—PSD95 in adolescent mice; Q/R—PSD95 in adult mice. (ZIP) [file pone.0136399.s002.zip › Figure S/Figure S1B - SYN 15d m2.tif]

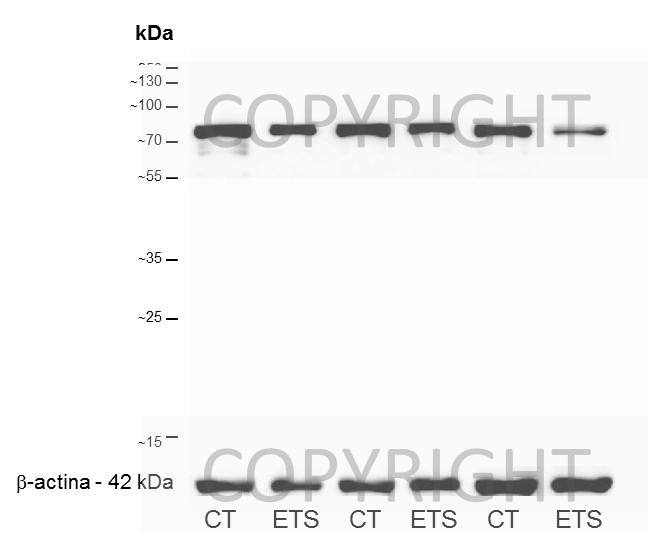

Supplement: S1 File — A/B—synapsin I in infant mice; C/D—synapsin I in adolescent mice; E/F—synapsin I in adult mice; G/H—synaptophysin in infant mice; I/J—synaptophysin in adolescent mice; K/L—synaptophysin in adult mice; M/N–PSD95 in infant mice; O/P—PSD95 in adolescent mice; Q/R—PSD95 in adult mice. (ZIP) [file pone.0136399.s002.zip › Figure S/Figure S1C - SYN 35d m1.tif]

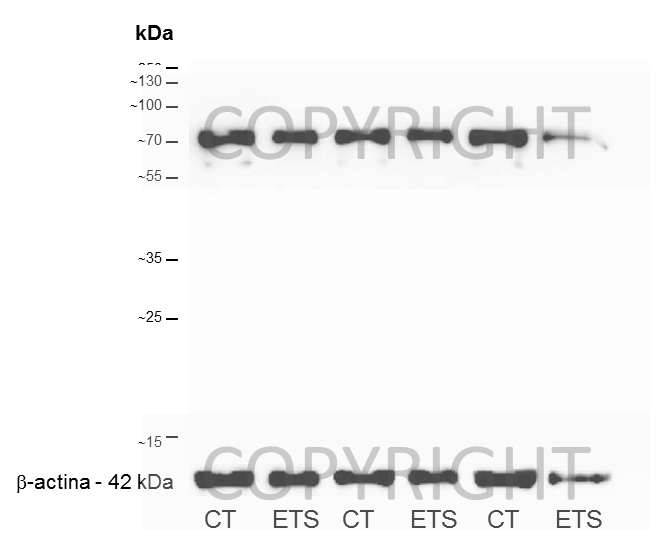

Supplement: S1 File — A/B—synapsin I in infant mice; C/D—synapsin I in adolescent mice; E/F—synapsin I in adult mice; G/H—synaptophysin in infant mice; I/J—synaptophysin in adolescent mice; K/L—synaptophysin in adult mice; M/N–PSD95 in infant mice; O/P—PSD95 in adolescent mice; Q/R—PSD95 in adult mice. (ZIP) [file pone.0136399.s002.zip › Figure S/Figure S1D - SYN 35d m2.tif]

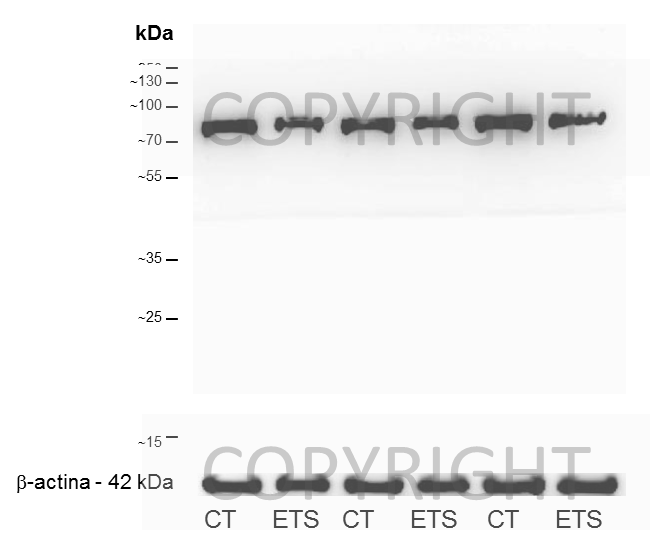

Supplement: S1 File — A/B—synapsin I in infant mice; C/D—synapsin I in adolescent mice; E/F—synapsin I in adult mice; G/H—synaptophysin in infant mice; I/J—synaptophysin in adolescent mice; K/L—synaptophysin in adult mice; M/N–PSD95 in infant mice; O/P—PSD95 in adolescent mice; Q/R—PSD95 in adult mice. (ZIP) [file pone.0136399.s002.zip › Figure S/Figure S1E - SYN 65d m1.tif]

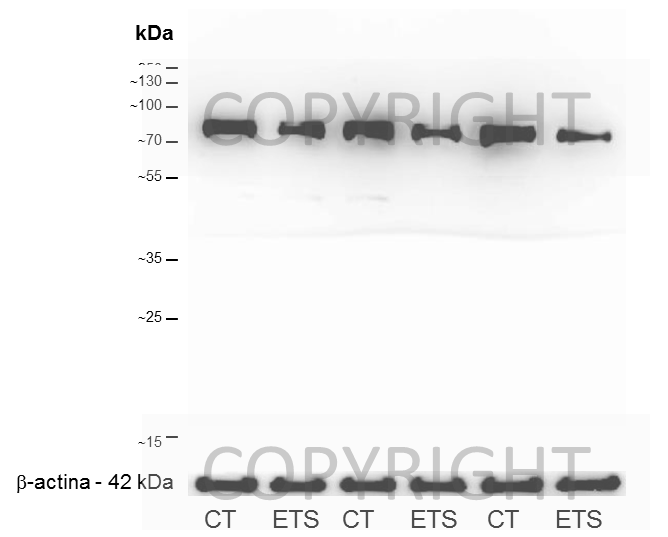

Supplement: S1 File — A/B—synapsin I in infant mice; C/D—synapsin I in adolescent mice; E/F—synapsin I in adult mice; G/H—synaptophysin in infant mice; I/J—synaptophysin in adolescent mice; K/L—synaptophysin in adult mice; M/N–PSD95 in infant mice; O/P—PSD95 in adolescent mice; Q/R—PSD95 in adult mice. (ZIP) [file pone.0136399.s002.zip › Figure S/Figure S1F - SYN 65d m2.tif]

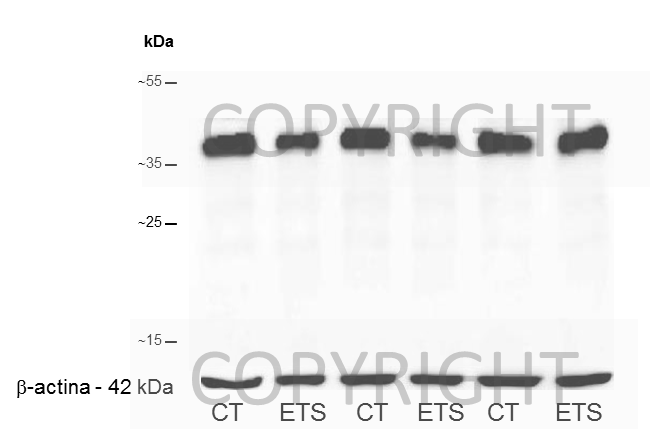

Supplement: S1 File — A/B—synapsin I in infant mice; C/D—synapsin I in adolescent mice; E/F—synapsin I in adult mice; G/H—synaptophysin in infant mice; I/J—synaptophysin in adolescent mice; K/L—synaptophysin in adult mice; M/N–PSD95 in infant mice; O/P—PSD95 in adolescent mice; Q/R—PSD95 in adult mice. (ZIP) [file pone.0136399.s002.zip › Figure S/Figure S1G - SYP 15d m1.tif]

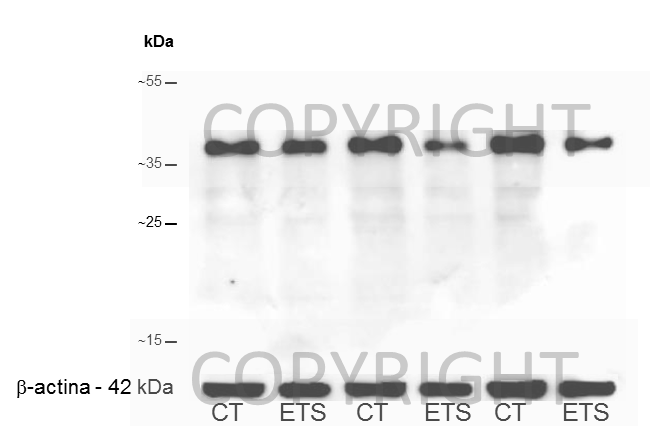

Supplement: S1 File — A/B—synapsin I in infant mice; C/D—synapsin I in adolescent mice; E/F—synapsin I in adult mice; G/H—synaptophysin in infant mice; I/J—synaptophysin in adolescent mice; K/L—synaptophysin in adult mice; M/N–PSD95 in infant mice; O/P—PSD95 in adolescent mice; Q/R—PSD95 in adult mice. (ZIP) [file pone.0136399.s002.zip › Figure S/Figure S1H - SYP 15d m2.tif]

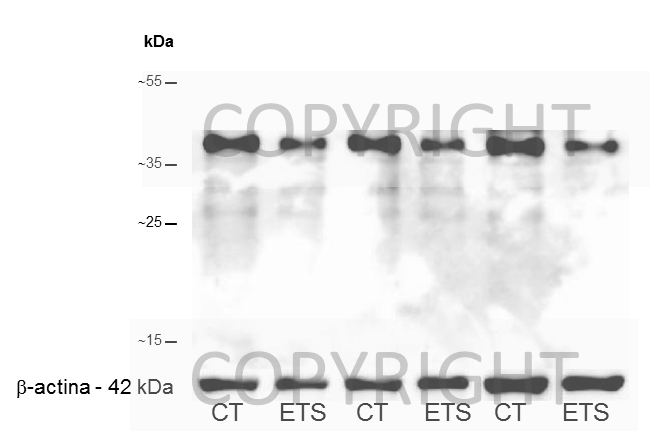

Supplement: S1 File — A/B—synapsin I in infant mice; C/D—synapsin I in adolescent mice; E/F—synapsin I in adult mice; G/H—synaptophysin in infant mice; I/J—synaptophysin in adolescent mice; K/L—synaptophysin in adult mice; M/N–PSD95 in infant mice; O/P—PSD95 in adolescent mice; Q/R—PSD95 in adult mice. (ZIP) [file pone.0136399.s002.zip › Figure S/Figure S1I - SYP 35d m1.tif]

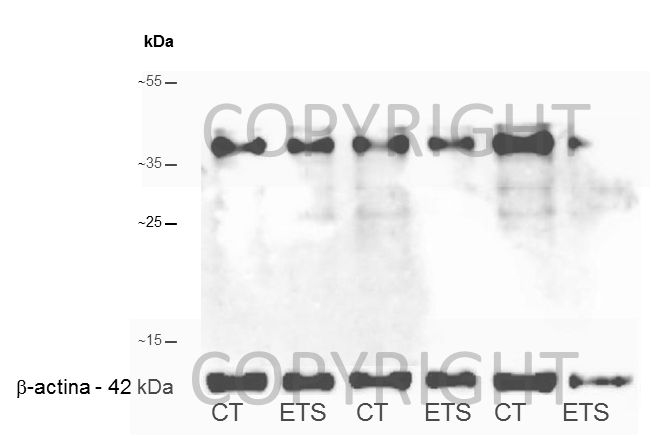

Supplement: S1 File — A/B—synapsin I in infant mice; C/D—synapsin I in adolescent mice; E/F—synapsin I in adult mice; G/H—synaptophysin in infant mice; I/J—synaptophysin in adolescent mice; K/L—synaptophysin in adult mice; M/N–PSD95 in infant mice; O/P—PSD95 in adolescent mice; Q/R—PSD95 in adult mice. (ZIP) [file pone.0136399.s002.zip › Figure S/Figure S1J - SYP 35d m2.tif]

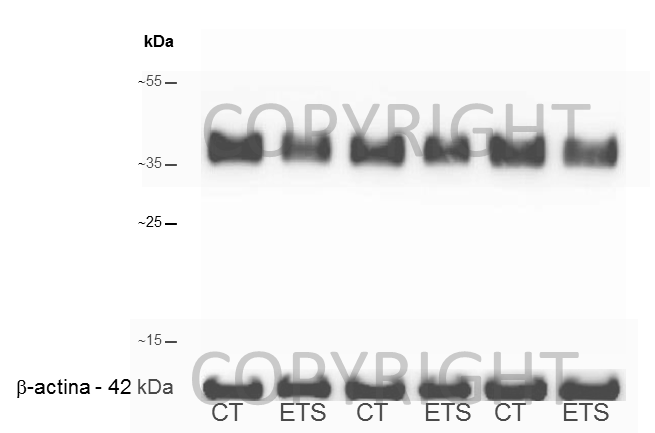

Supplement: S1 File — A/B—synapsin I in infant mice; C/D—synapsin I in adolescent mice; E/F—synapsin I in adult mice; G/H—synaptophysin in infant mice; I/J—synaptophysin in adolescent mice; K/L—synaptophysin in adult mice; M/N–PSD95 in infant mice; O/P—PSD95 in adolescent mice; Q/R—PSD95 in adult mice. (ZIP) [file pone.0136399.s002.zip › Figure S/Figure S1K - SYP 65d m1.tif]

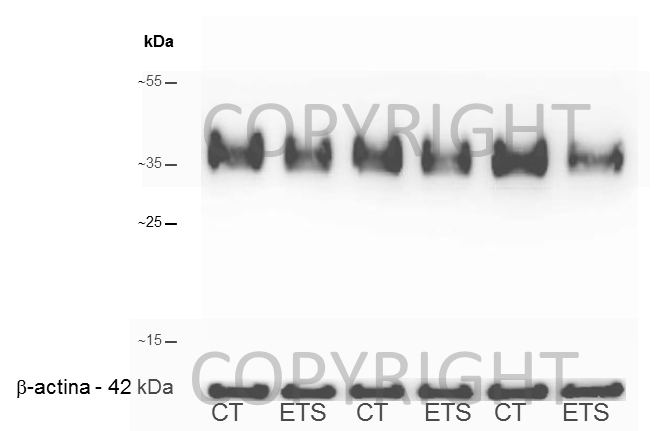

Supplement: S1 File — A/B—synapsin I in infant mice; C/D—synapsin I in adolescent mice; E/F—synapsin I in adult mice; G/H—synaptophysin in infant mice; I/J—synaptophysin in adolescent mice; K/L—synaptophysin in adult mice; M/N–PSD95 in infant mice; O/P—PSD95 in adolescent mice; Q/R—PSD95 in adult mice. (ZIP) [file pone.0136399.s002.zip › Figure S/Figure S1L - SYP 65d m2.tif]

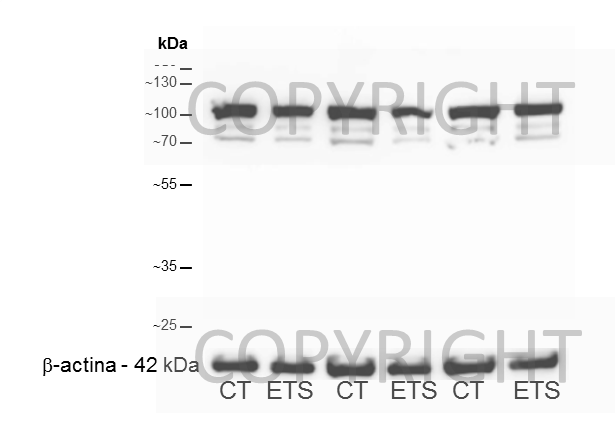

Supplement: S1 File — A/B—synapsin I in infant mice; C/D—synapsin I in adolescent mice; E/F—synapsin I in adult mice; G/H—synaptophysin in infant mice; I/J—synaptophysin in adolescent mice; K/L—synaptophysin in adult mice; M/N–PSD95 in infant mice; O/P—PSD95 in adolescent mice; Q/R—PSD95 in adult mice. (ZIP) [file pone.0136399.s002.zip › Figure S/Figure S1M - PSD95 15d m1.tif]

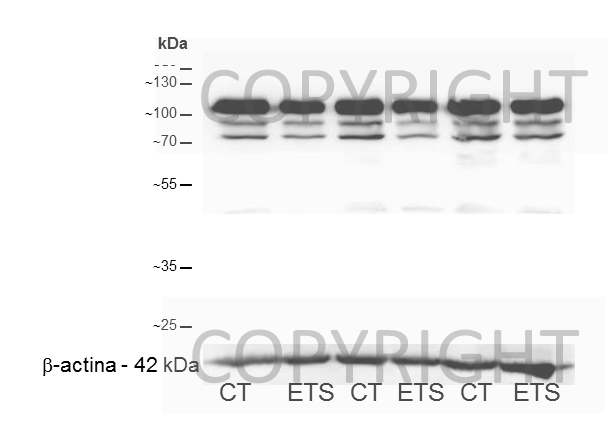

Supplement: S1 File — A/B—synapsin I in infant mice; C/D—synapsin I in adolescent mice; E/F—synapsin I in adult mice; G/H—synaptophysin in infant mice; I/J—synaptophysin in adolescent mice; K/L—synaptophysin in adult mice; M/N–PSD95 in infant mice; O/P—PSD95 in adolescent mice; Q/R—PSD95 in adult mice. (ZIP) [file pone.0136399.s002.zip › Figure S/Figure S1N - PSD95 15d m2.tif]

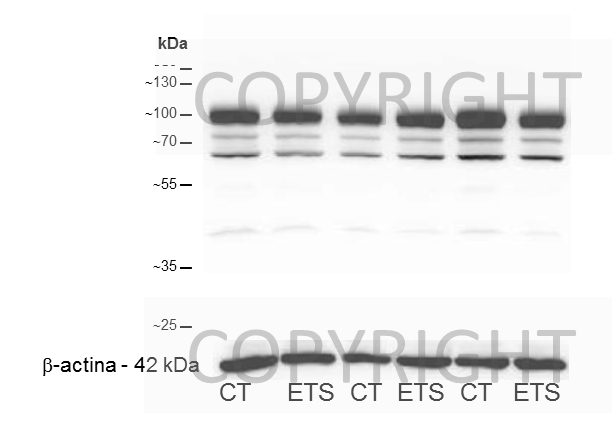

Supplement: S1 File — A/B—synapsin I in infant mice; C/D—synapsin I in adolescent mice; E/F—synapsin I in adult mice; G/H—synaptophysin in infant mice; I/J—synaptophysin in adolescent mice; K/L—synaptophysin in adult mice; M/N–PSD95 in infant mice; O/P—PSD95 in adolescent mice; Q/R—PSD95 in adult mice. (ZIP) [file pone.0136399.s002.zip › Figure S/Figure S1O - PSD95 35d m1.tif]

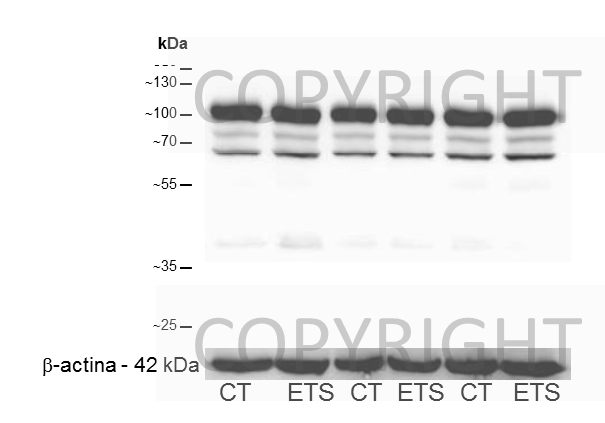

Supplement: S1 File — A/B—synapsin I in infant mice; C/D—synapsin I in adolescent mice; E/F—synapsin I in adult mice; G/H—synaptophysin in infant mice; I/J—synaptophysin in adolescent mice; K/L—synaptophysin in adult mice; M/N–PSD95 in infant mice; O/P—PSD95 in adolescent mice; Q/R—PSD95 in adult mice. (ZIP) [file pone.0136399.s002.zip › Figure S/Figure S1P - PSD95 35d m2.tif]

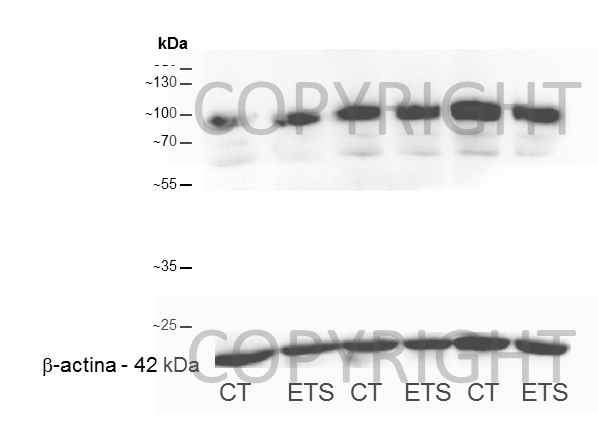

Supplement: S1 File — A/B—synapsin I in infant mice; C/D—synapsin I in adolescent mice; E/F—synapsin I in adult mice; G/H—synaptophysin in infant mice; I/J—synaptophysin in adolescent mice; K/L—synaptophysin in adult mice; M/N–PSD95 in infant mice; O/P—PSD95 in adolescent mice; Q/R—PSD95 in adult mice. (ZIP) [file pone.0136399.s002.zip › Figure S/Figure S1Q - PSD95 65d m1.tif]

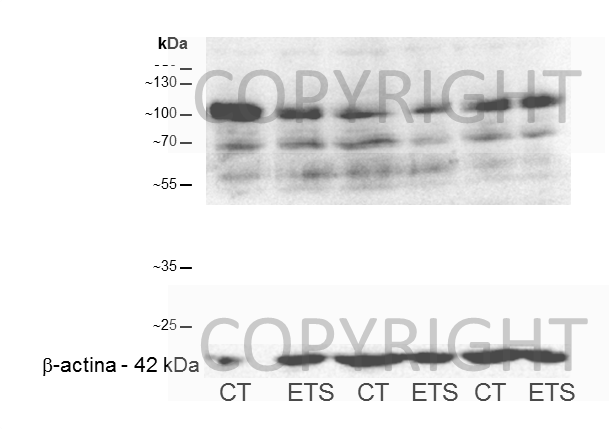

Supplement: S1 File — A/B—synapsin I in infant mice; C/D—synapsin I in adolescent mice; E/F—synapsin I in adult mice; G/H—synaptophysin in infant mice; I/J—synaptophysin in adolescent mice; K/L—synaptophysin in adult mice; M/N–PSD95 in infant mice; O/P—PSD95 in adolescent mice; Q/R—PSD95 in adult mice. (ZIP) [file pone.0136399.s002.zip › Figure S/Figure S1R - PSD95 65d m2.tif]
